# Characterization of Heterotopic Ossification Using Radiographic Imaging: Evidence for a Paradigm Shift

**DOI:** 10.1371/journal.pone.0141432

**Published:** 2015-11-06

**Authors:** R. Cameron Brownley, Shailesh Agarwal, Shawn Loder, Oluwatobi Eboda, John Li, Joshua Peterson, Charles Hwang, Christopher Breuler, Vesa Kaartinen, Bin Zhou, Yuji Mishina, Benjamin Levi

**Affiliations:** 1 Department of Surgery, University of Michigan, Ann Arbor, MI, United States of America; 2 School of Dentistry, University of Michigan, Ann Arbor, MI, United States of America; 3 Albert Einstein School of Medicine, New York, NY, United States of America; University of Zaragoza, SPAIN

## Abstract

Heterotopic ossification (HO) is the growth of extra-skeletal bone which occurs following trauma, burns, and in patients with genetic bone morphogenetic protein (BMP) receptor mutations. The clinical and laboratory evaluation of HO is dependent on radiographic imaging to identify and characterize these lesions. Here we show that despite its inadequacies, plain film radiography and single modality microCT continue to serve as a primary method of HO imaging in nearly 30% of published *in vivo* literature. Furthermore, we demonstrate that detailed microCT analysis is superior to plain film and single modality microCT radiography specifically in the evaluation of HO formed through three representative models due to its ability to 1) define structural relationships between growing extra-skeletal bone and normal, anatomic bone, 2) provide accurate quantification and growth rate based on volume of the space-occupying lesion, thereby facilitating assessments of therapeutic intervention, 3) identify HO at earlier times allowing for evaluation of early intervention, and 4) characterization of metrics of bone physiology including porosity, tissue mineral density, and cortical and trabecular volume. Examination of our trauma model using microCT demonstrated two separate areas of HO based on anatomic location and relationship with surrounding, normal bone structures. Additionally, microCT allows HO growth rate to be evaluated to characterize HO progression. Taken together, these data demonstrate the need for a paradigm shift in the evaluation of HO towards microCT as a standard tool for imaging.

## Introduction

Heterotopic ossification (HO) is the pathologic development of extra-skeletal bone which develops after severe burns, musculoskeletal trauma, and spinal cord injury. Separately, patients with a genetic mutation in the type I bone morphogenetic protein (BMP) receptor *ACVR1* develop ectopic bone lesions after minimal trauma to the soft tissues[1]. All variants of HO present a significant burden to patients due to persistent pain, poor wound healing, decreased range of motion (ROM) at affected joints, and increased risk of nerve entrapment[2,3].

Identification of HO presents a substantial clinical challenge as symptoms often precede radiologic evidence of ectopic bone. This is a critical point, as there is currently no effective medical treatment for this condition and definitive surgical resection is limited by high rates of recurrence[3–7].

In the laboratory, several animal models have been developed to study HO. In mice, HO has been induced using hind limb tendon transection, mutants with *Cre-*driven constitutively active *ACVR1* expression, mutants with Ad.cre induction of constitutively active *ACVR1* expression with concomitant muscle injury (Ad.cre/cardiotoxin), and BMP-impregnated scaffolds[3,8–11]. These models have been interrogated to assess the cellular and histologic changes which take place during HO formation. Imaging of these models is also performed routinely to obtain gross morphologic data regarding HO location and size, and response to therapeutic interventions.

High-quality radiographic imaging is a cornerstone of bone research. Through microCT scan technology, one can quantify total bone volume, and also quantify bone density, differences in cortical thickness and trabecular volume, and even quantify progression over time[12–18]. These characteristics are particularly important as HO lesions may develop in proximity to normal bone, which can obscure identification and delineation from normal surrounding structures. From 2004 to 2014, the number of published HO studies has more than doubled (2004: 183 studies vs. 2014: 402 studies). Despite this, a large percentage of HO studies continue to utilize plain film radiographic imaging as the primary method of imaging animal models. Obtaining more detailed imaging is vital for a precise assessment of therapeutic interventions.

In this study, we hypothesized that the currently available literature continues to utilize plain film radiography and single modality microCT analysis in HO which is inadequate. We further demonstrate the specific differences between plain film and microCT imaging using three distinct models of HO: musculoskeletal trauma induced with local tendon transection and burn injury (burn/tenotomy model), intramuscular Ad.cre and cardiotoxin injection in a *caACVR1* ^*fx/fx*^ mouse (Ad.cre/cardiotoxin-induced model), and *Cre-*driven constitutively active *ACVR1* expression (genetic model). Most importantly, we further characterize and describe the growth rate and bone characteristics of HO in several key models which are used to study genetic and trauma induced forms of HO. Our objective is to provide convincing evidence that microCT analysis should be a cornerstone of HO analysis in the laboratory to assess extra-skeletal bone development as well as the efficacy of treatment strategies.

## Materials and Methods

### Literature search

Murine HO studies were identified using PubMed search for heterotopic ossification (title) and either murine, rat, or mouse in all fields. Only those studies published from 2005 onwards were included. Review manuscripts were excluded, as were studies which did not perform any radiographic HO imaging (*e*.*g*. studies of progenitor cell populations).

### Ethics Statement

All animal experiments described were approved by the University Committee on Use and Care of Animals at the University of Michigan-Ann Arbor (Protocols: #05909, 05182, and 05716). This study was carried out in strict accordance with the recommendations in the Guide for the Care and Use of Laboratory Animals of the National Institutes of Health. All animal procedures were carried out in accordance with the guidelines provided in the *Guide for the Use and Care of Laboratory Animals*: *Eighth Edition* from the Institute for Laboratory Animal Research (ILAR, 2011).

### Animals

Mice included for extra-skeletal bone evaluation were wild type C57BL/6 (Charles River Laboratory), *caAcvr1* ^*fl*:*fl*^, *Nfatc1-Cre/caAcvr1* ^*fl*:*wt*^, or littermate controls[8,19]. All breeding was performed at the University of Michigan in facilities managed by the Unit for Laboratory Animal Medicine (ULAM) at the University of Michigan. Tail genomic DNA was used for genotyping.

### Extra-skeletal bone models

Burn/tenotomy mice received a 30% total body surface area (TBSA) partial-thickness burn on the shaved dorsum followed by left hindlimb Achilles’ tendon transection. All burn/tenotomy mice (n = 6) received pre-surgical analgesia consisting of 0.1 mg/kg buprenorphine, followed by anesthesia with inhaled isoflurane, and close post-operative monitoring with analgesic administration. The dorsum was burned using a metal block heated to 60C in a water bath and applied to the dorsum for 18 seconds continuously. The tenotomy site was closed with a single 5–0 vicryl stitch placed through the skin only.

Burn/tenotomy mice were treated with local injections of apyrase (n = 3) or celecoxib (n = 3) to injury site in the manner previously described[3].

*caAcvr1* ^*fl*:*fl*^ mice received hindlimb cardiotoxin and Ad.cre injection 24 days after birth. Mice were then euthanized 17 (n = 3) and 22 days (n = 6) after cardiotoxin and Ad.cre injection.

*Nfatc1-Cre/caAcvr1* ^*fl*:*wt*^ mice were generated by crossing *Nfatc1-Cre* ^*+*^ mice with *caAcvr1* ^*fl*:*wt*^ mice. Resulting mutants developed extra-skeletal bone by 4–5 days after birth and underwent hindlimb analyses at P11 (n = 2), 20 (n = 2), 30 (n = 4), and 40 (n = 2) days after birth. Corresponding littermate controls were analyzed at the same time points.

### Plain film radiographic imaging

Live mice used for X-rays were anesthetized with isofluorine before placement into faxitron (Faxitron x-ray corporation). All limbs were imaged for 10 seconds at 3x distance and KV = 23. Radiographic film was developed by standard measures (Hope Micro-Max) before scanning for digital formatting. Digital images obtained were cropped and adjusted for optimal brightness and contrast in Adobe Photoshop CS5.

### Plainfilm radiographic quantification

Radiographic film was scanned for digital analysis in Adobe Photoshop CS5. The magnetic lasso tool was used to encircle regions of HO in mouse hindlimbs. Total pixel area was calculated in each region and multiplied by the average luminosity value to obtain volume measurement (pixels x mean luminosity).

### MicroCT imaging

Live mice were anesthetized with isofluorine before and during microCT scanning. The Siemens Inveon (University of Michigan Center for Molecular Imaging Core) utilized an 80 W tungsten anode to consistently image animals and samples in a 10x10 cm field of view at a 48 micron resolution (80 kVP, 80 mA, and 1,100 ms exposure). Animal body temperature was maintained by scanner equipped heating pad over 15–20 minute intervals.

### MicroCT file reformatting

GEHC Microview software was used for importing raw data into compatible “.vff” file used for subsequent analysis (Import Settings: X spacing: 0.04804, Y spacing: 0.04804, Z spacing: 0.04804; Data type = short; Data Endian: little endian; X size: 800, Y size: 800, Z size: 800). Initial “.vff” file was then condensed to desired region of interest in GEHC Sliceview before reorientation on GEHC Microview to align z plane along marrow space of the tibia. Reoriented file size was then condensed once again in GEHC Sliceview before visual imaging and quantification on GEHC Microview.

### MicroCT 3D Reconstruction

**Fig 1:**
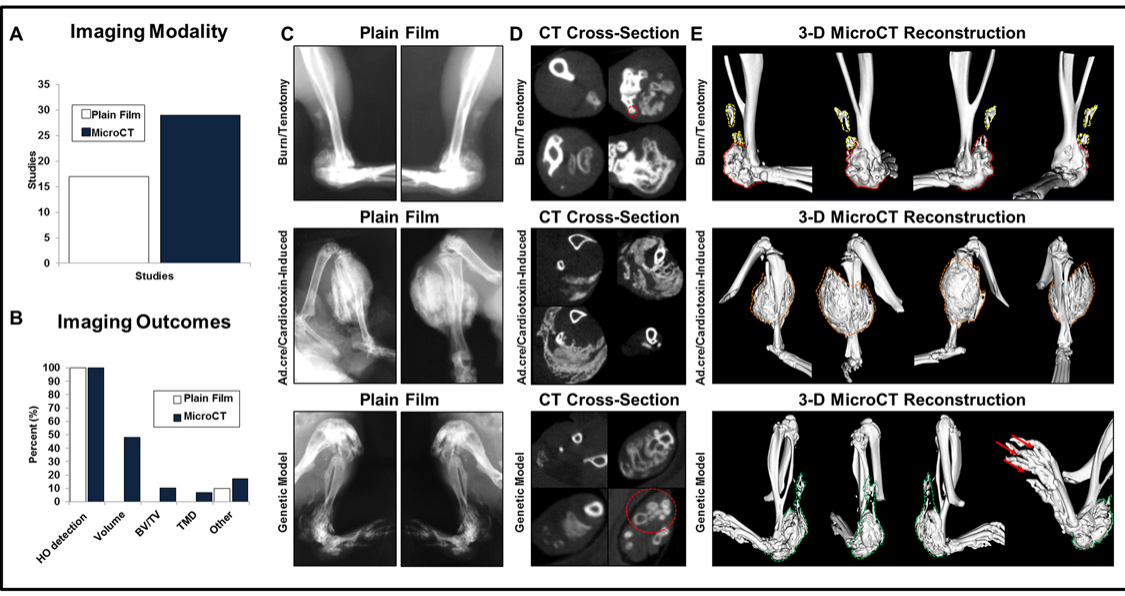
Literature review of current HO imaging modalities and the role of positioning on HO identification in three experimental models. (A) Comparison of plain film (n = 17; 43%) and microCT (n = 29; 73%) radiographic imaging in published manuscripts studying HO between 2005–2015. (B) Imaging outcomes from HO studies using plain film radiographs vs. microCT. (C) Plain film radiographs of HO in two separate orientations for burn/tenotomy (9 weeks after injury), Ad.cre/cardiotoxin-induced (17 days post-injection), and genetic (day 50) models. (D) MicroCT cross-sections demonstrating areas of HO in burn/tenotomy, Ad.cre/cardiotoxin-induced, and genetic HO models. (E) 3D microCT reconstructions (800 HU) of HO sites with 90° clockwise rotations of same limb in all 3 HO models (*red*: bone-associated HO; *yellow*: soft tissue HO; *orange*: Ad.cre/cardiotoxin-induced HO; *green*: genetic HO; *red arrows*: HO between toes).

**Fig 2:**
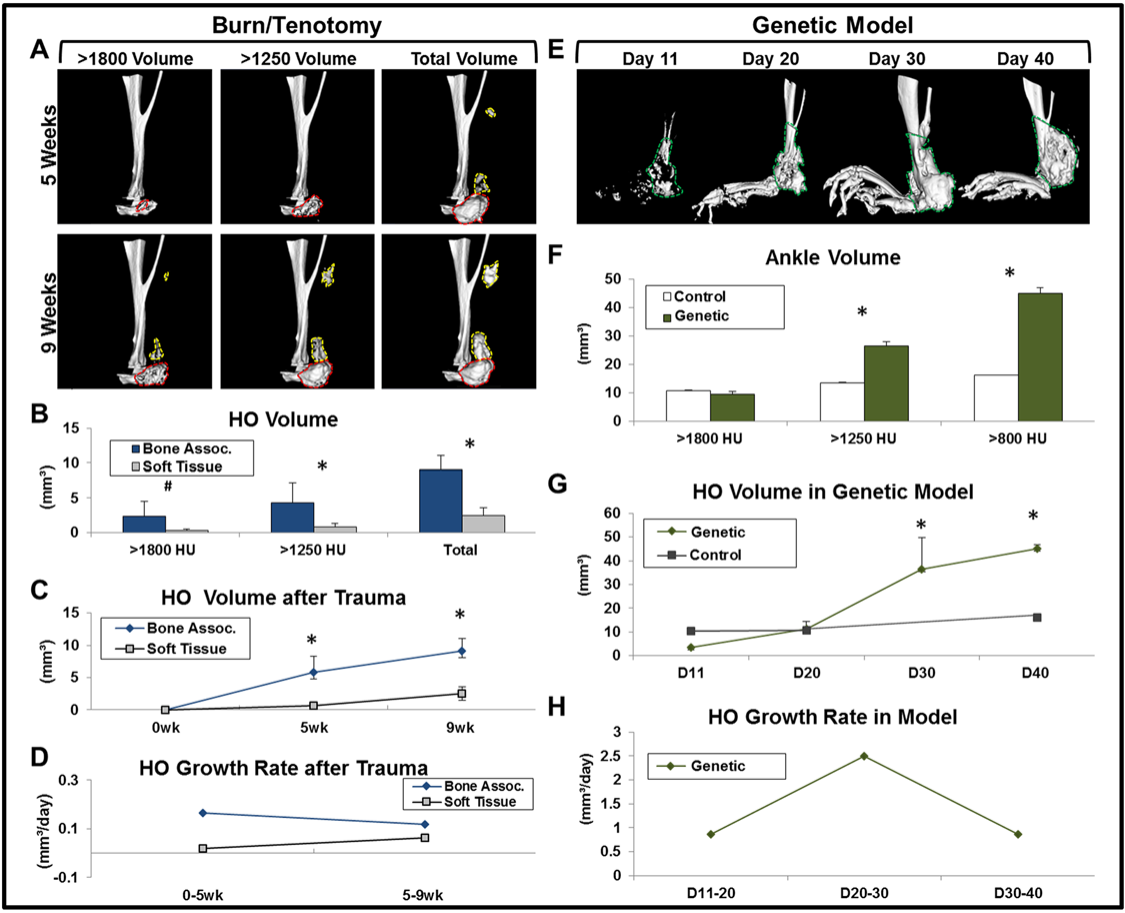
MicroCT allows for superior volume and growth quantification of HO. (A) Representative 3D reconstructions of normal, anatomic bone, bone-associated HO *(red outline*), and soft tissue HO (*yellow outline*) of burn/tenotomy model at 5 and 9 weeks; HO visual rendered at three density-dependent thresholds. (B) Total and density-dependent volumes of HO in burn/tenotomy model at 9 weeks after injury. (C) HO volume following trauma in two separate anatomic sites (bone-associated or soft tissue) at separate time points. (D) Mean daily growth rate of HO in burn/tenotomy model based on longitudinal, total volume quantification, (E) Representative 3D reconstructions (800 HU) of normal, anatomic bone and genetic HO (*green outline*) at four timepoints. (F) Density-dependent volumes of day 40 genetic HO model. (G) Genetic HO 800HU volume at separate timepoints. (H) Mean daily growth rate of HO in genetic model based on longitudinal, 800HU volume quantification. (In graphs: * = p<0.05; # = p<0.07).

The “Advanced ROI Tool” (GEHC Microview) was used to create contours within a 50 cc conical tube filled with water and through air. Next, 3-dimensional regions of interest (3D ROI’s) were generated and used to attain the mean Arbitrary Density Unit (ADU) of each ROI (Basic Bone Analysis, GEHC Microview). These mean values were then input into a calibration equation (*i*.*e*. ADU_800 HU_ = m*800+b where m = (ADU_air_-ADU_water_)/-100 and b = ADU_air_-(m*-1000)) on Microsoft Excel to attain equivalent ADU values for -500, 800, 1250, 1800, and 3420 Hounsfield unit densities. Manual contours were drawn around the hindlimb in each HO model and were visualized (Visualize>Isosurface>Image Threshold: ADU_1800 HU_ or ADU_800HU_; Surface Quality Factor: 1.00) (Fig 1C, Fig 2A and 2D). For imaging 1800HU, 1250HU, and total volumes of HO growth, the ADU_1800HU_, ADU_1250_, and ADU_-500HU_ were used as threshold inputs to visualize contours generated around HO sites (Fig 2A).

### MicroCT quantification

**Fig 3:**
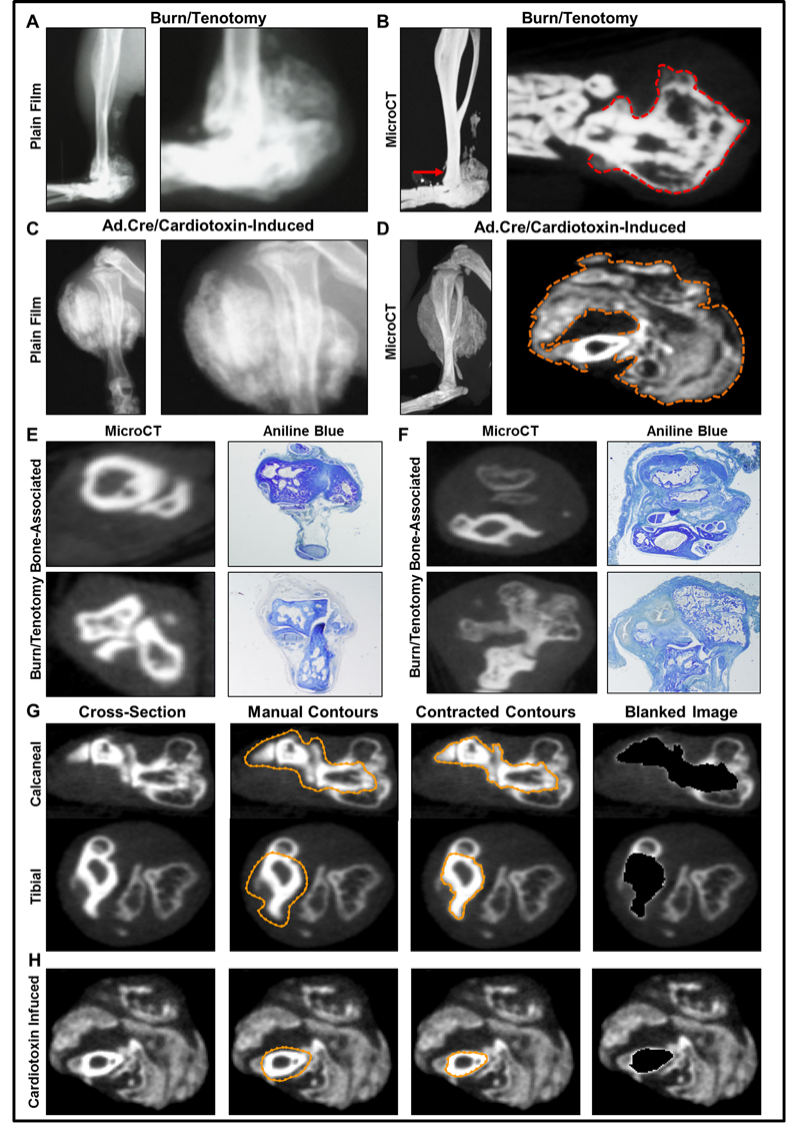
MicroCT allows clear delineation of HO from normal anatomic bone. **(**A) Plain film radiograph of 9-week burn/tenotomy of mouse hindlimb to demonstrate obscuring effect by normal anatomic bone. (B) High resolution microCT image of burn/tenotomy mouse hindlimb 9 weeks post-op (*red arrow*: axial plane of cross section; *red outline*: *bone-associated HO growth at calcaneus*). (C) Plain film radiograph of day 22 Ad.cre/cardiotoxin-induced mouse hindlimb to demonstrate obscuring effect by normal anatomic bone (D) High resolution microCT image of day 22 Ad.cre/cardiotoxin-induced hindlimb with representative serial cross sections (*orange outline*: Ad.cre/cardiotoxin-induced HO). (E) Histologic cross sections of uninjured, contralateral mouse hindlimb stained with aniline blue with comparable microCT cross sections depicting normal tibia, talus, and calcaneal bones. (F) Experimental burn/tenotomy hindlimb histologic cross sections stained with aniline blue with comparable microCT cross sections depicting normal tibia, talus, and calcaneal bones with HO sites. (G) MicroCT evaluation of HO with blanking technique demonstrated to remove normal bone (*orange dotted circles*) from cross-sections of burn/tenotomy model. H) MicroCT evaluation of HO with blanking technique demonstrated to remove normal bone (*orange dotted circles*) from cross-sections of Ad.cre/cardiotoxin-induced model.

**Fig 4:**
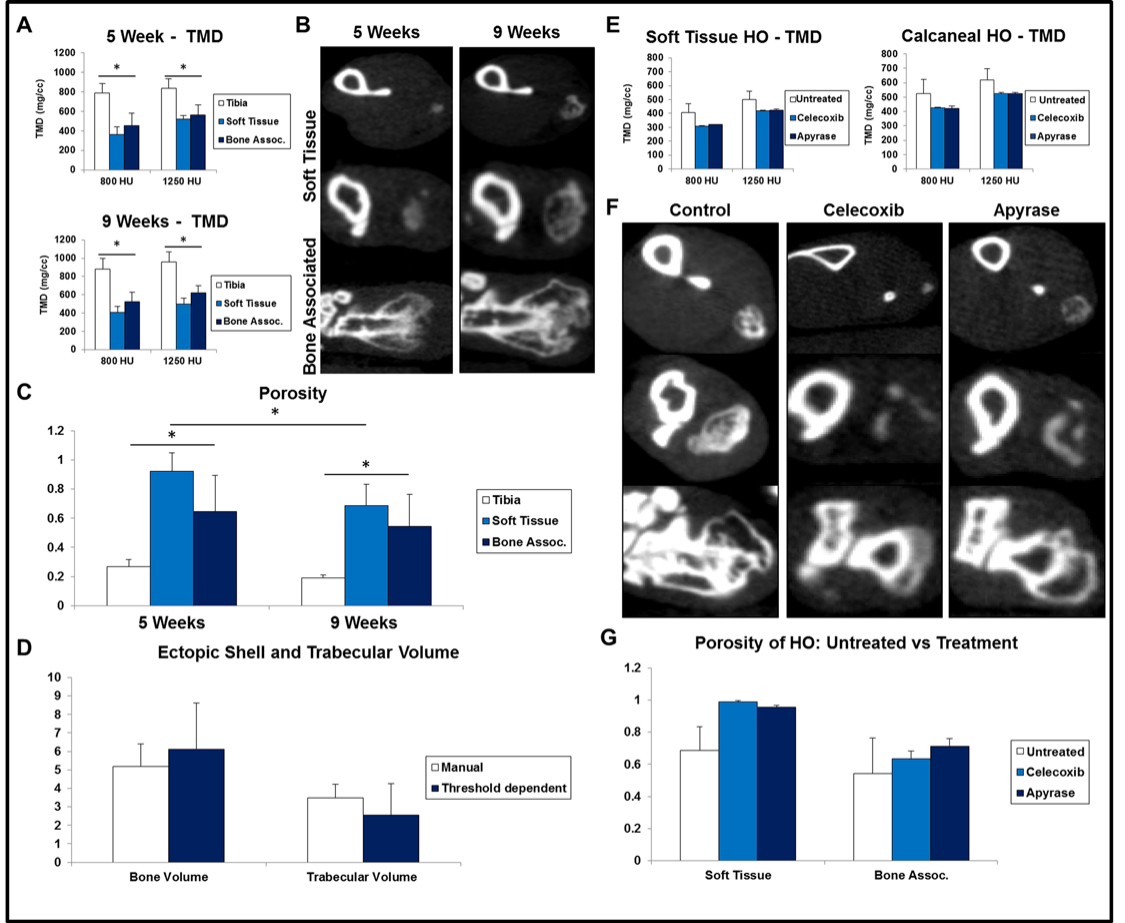
MicroCT can evaluate HO metrics in addition to volume. *(*A) Average tissue mineral density (TMD) of tibia, soft tissue HO and bone-associated HO evaluated at 5 and 9 weeks post-op in burn/tenotomy model. (B) Representative cross sectional images of soft tissue HO (*top two rows*) and bone-associated HO (*bottom row*) at 5 and 9 weeks in burn/tenotomy model. (C) Tibial bone, soft tissue HO, and bone-associated HO in burn/tenotomy model evaluated for average porosity using 1250 HU threshold. (D) Burn/tenotomy HO shell and marrow space average volumes isolated with manual, trabecular contours (*white*) or by automated 800 HU threshold (*blue*). (E) Average TMD of soft tissue and bone-associated HO in burn/tenotomy mice that were: untreated, celecoxib-injected, or apyrase-injected. (F) Representative cross sectional images of soft tissue HO (*top two rows*) and bone-associated HO (*bottom row*) of untreated and treated burn/tenotomy groups at 9 weeks after injury. (G) Average porosity of soft tissue (*left*) and calcaneal (*right*) HO in burn/tenotomy untreated and treated groups.

To separate native bone from HO, the ADU_1800 HU_ value was used (GEHC Microview, Advanced ROI, Advanced Options, Threshold: ADU_1800 HU_; Resolution: 5 nodes/10px) to contract polygonal contours along the user-determined regions of tibia, talus, and calcaneus. These contours were then used to generate a 3D ROI for normal bone which was subsequently blanked out of the scan (Fig 3G and 3H). Spline contours were manually drawn around sites of HO and respective 3D ROI’s were generated. Next, the “Basic Bone Analysis” tool (GEHC Microview) was calibrated (*Advanced Options*: Bone ADU: ADU_3420 HU_, Water ADU: ADU_water_, Lower Exclusion ADU: ADU_-500 HU,_ Upper Exclusion ADU: 65,545). In order to quantify HO volume based on the tissue’s density, the ADU_-500_, ADU_1800_, ADU_1250_ values were used for threshold inputs (Fig 2B and 2F). Tissue mineral density (TMD) refers to a mass of bone matrix with specified density (in Hounsfield units) that is divided by its volume and was also calculated using the “Bone Analysis” tool (ADU_1250HU_ and ADU_800HU_ threshold inputs) (Fig 4A and 4E). The porosity ratio was calculated in Microsoft Excel using the following equation: Porosity = 1-(Volume_1250 HU_ / Volume_total_) (Fig 4C and 4G). Finally, ectopic shell and trabecular volumes were calculated manually (using ADU_-500_ input) through generation of total and trabecular HO ROI’s (with difference being ectopic shell volume) and automatically, using ADU_1250_ input for ectopic shell volume (with total volume difference being trabecular volume) (Fig 4D).

### Histological Staining

Mice were euthanized and confirmed dead with cervical dislocation at late timepoints before limbs were harvested. After 24 hour fixation in formaldehyde at 4°C, limbs were placed in EDTA decalcification solution for 3–4 weeks at 4°C. Tissue was then dehydrated, and preserved with xylene via an automated 48-hour bone tissue program (Leica ASP 300 paraffin tissue processor, University of Michigan Microscopy and Image Analysis Core). Limb samples were then paraffin embedded (Leica EG1150 C), longitudinally sectioned (Leica RM2255) and slided. Sections were rehydrated and stained with aniline blue reagents to histologically identify bone cortex.

### Statistical Analysis

Means and standard deviations (s.d.) were calculated from numerical data, as presented in the text, figures, and figure legends. In figures, bar graphs represent means, whereas error bars represent one s.d. Statistical analysis was performed using an appropriate analysis of variance when more than two groups were compared, followed by a post hoc Student’s t test (with a Bonferroni correction) to directly compare two groups. Inequality of s.d. was excluded by using the Levene’s test. P values are included in the figure legends. P <0.05 was defined as the threshold for statistical significance. Statistical analysis was blinded.

## Results

### Plain film and single modality MicroCT are primarily used to study heterotopic ossification in animal models

To illustrate existing need for more robust imaging modalities in HO research, we first examined the literature published within the past 10 years studying HO in animal models. We found a total of 39 studies which were included for analysis based on our search criteria (S1 Appendix). Radiographic imaging methods and recorded outcomes obtained based on imaging modality were noted (Fig 1A and 1B). Plain film radiography was often used (n = 17 studies; 43%) as a method to confirm the gross presence or absence of HO. However, additional bone measurements were only found in 1 study (6%) in those using this modality. In the studies that utilized microCT for imaging (n = 29 studies; 74%), the technique’s primary use was for HO detection and to provide a visual display confirming observations. Only 14 (35%) of all studies found in our search quantified HO volume. Less were able to measure other bone characteristics such as bone volume fraction (BV/TV), tissue mineral density (TMD), or other widely utilized bone measurements (*i*.*e*. trabecular measurements).

### Plain film radiographic imaging is dependent on animal positioning

Plain film radiographs provide a 2-dimensional image of the 3-dimensional extra-skeletal bone. Thus, we hypothesized that plain film imaging would yield different images based on the orientation of the animal. Plain film radiography was performed on mice from our burn/tenotomy, Ad.cre/cardiotoxin-induced, and genetic HO models to obtain images of each mouse in 2 separate orientations (Fig 1C). As might be expected, we found that the orientation of the animal substantially changed the structural appearance of HO. Additionally, HO growth was often obscured by normal bone structures in the film, despite imaging in multiple orientations. After scanning the same mice with microCT, we noted that 2-dimensional, cross-section renditions provided clear visual data that diminished the obscuring effects found in x-ray film (Fig 1D). Furthermore, 3-dimensional reconstructions rendered from multiple orientations allowed for an appreciation of the external HO structure (Fig 1E). The synthesis of 2-dimensional cross-section data and 3-dimensional reconstructed images allowed for the identification of several distinct observations in each model. In the burn/tenotomy model, we identified a discrete region of HO growth forming in proximity to the lateral malleolus; this was not identifiable in plain film radiographs due to the underlying normal anatomic, malleolar bone (Fig 1E, top row, red outline). In the Ad.Cre/Cardiotoxin model, use of cross sections allows for the separation of normal anatomic bone from the HO (Fig 1E, middle row). This is discussed further below. Additionally, the curved toe phenotype in our genetic model was much clearer, confirming low-density bone forming between the digits (Fig 1E, bottom row).

### Plain film radiography is unable to delineate HO from adjacent, normal bone

A major challenge in the evaluation of ectopic bone is the physical delineation of extra skeletal from normal bone. HO may form in juxtaposition to normal bone–in these situations it is unclear whether the ectopic bone represents a separate entity or a continuous extension of normal skeletal bone. We hypothesized that unlike plain film radiography, microCT imaging adequately allows for delineation of ectopic bone from adjacent, normal bone. When burn/tenotomy and Ad.cre/cardiotoxin-induced mice underwent plain film imaging, irregularities along the normal bone structures were observed (Fig 3A and 3C). However, it was not possible to determine the border of the normal bone and the growing pathologic bone. When microCT was performed on these same hindlimbs, serial cross-sections allowed for clear delineation of the extra-skeletal bone (Fig 3B and 3D). Histological staining was performed on the same burn/tenotomy hindlimb, revealing normal-ectopic bone boundaries that corresponded with microCT cross sections (Fig 3E and 3F). Finally, we show that normal bone can actually be encircled by a series of contours and completely removed from the microCT viewing plane, allowing for a complete isolation of ectopic bone (Fig 3G and 3H). This blanking analysis method greatly reduces the possibility of user error when defining sites of HO, enabling quantification of bone-associated HO in a standardized and reproducible manner.

### MicroCT allows for volume quantification of HO

Discovery of therapeutic agents to treat HO is of substantial interest, necessitating quantitative measures which can adequately assess the impact of these agents on HO. Since plain film radiographs are orientation-dependent, with regular bone structures that overlap the HO, measurement can be difficult and inconsistent. Next, to demonstrate that microCT can be used to quantify HO in a standardized manner, 3-dimensional regions of interest (3D ROI’s) were created within microCT scans of our burn/tenotomy and genetic HO models (Fig 2A and 2E). Subsequent analysis of these 3D ROI’s was used to calculate HO volume across multiple ranges of density (Fig 2B and 2F). We found that bone-associated HO in our burn/tenotomy model was significantly higher at 1250HU and total volumes (p<0.05). In the genetic model, ankle bone volume was significantly greater than littermate controls at 1250HU and 800HU volumes (p = 0.05 and p = 0.027). When additional timepoint values were added to each data set, HO time courses were produced to show HO volume progression (Fig 2C and 2G). Further analysis of these values enabled us to calculate the growth rates for HO in two distinct anatomic locations of the burn/tenotomy model (Fig 2D). The same analysis was applied to the genetic model, clarifying a time interval of maximum growth (Fig 2H). As a whole, this data suggests that HO can be accurately and reproducibly quantified in a manner that is objective and efficient.

### MicroCT can identify ectopic bone at earlier time points when compared with x-ray

**Fig 5:**
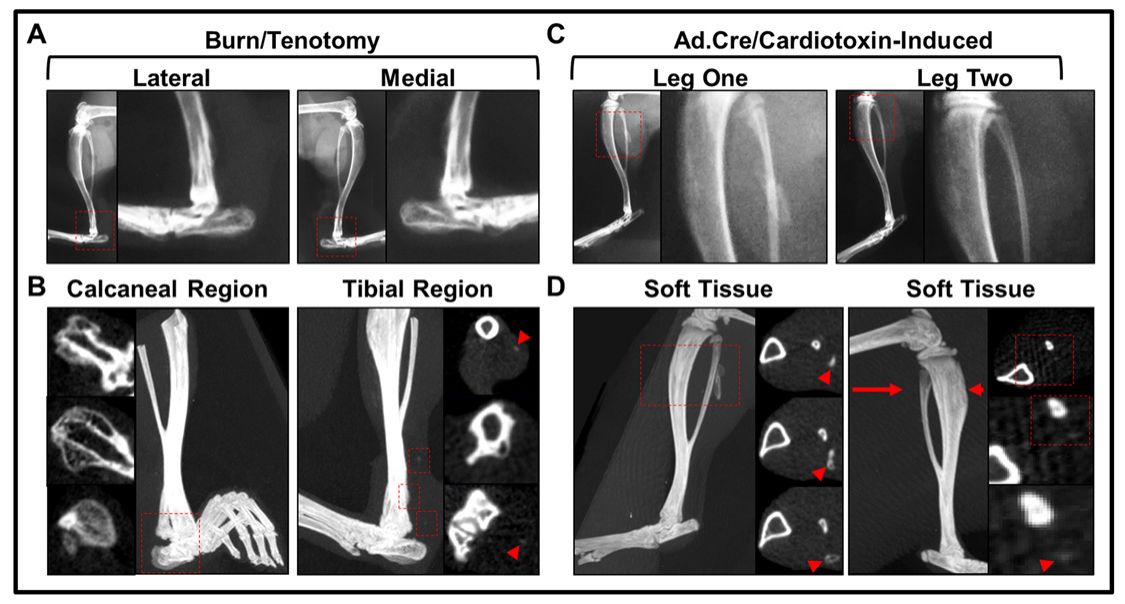
MicroCT allows for earlier identification of HO. (A) Plain film radiographs taken of injured limb in burn/tenotomy model 24 days after injury (*red boxes*: zoomed region of high risk HO site). (B) High resolution microCT image of injured limb and representative cross sections depicting mineral deposition in calcaneal (bone-associated) (*left images*) and tibial (*right images*) regions of burn/tenotomy mouse 24 days after injury. (C) Plain film radiographs taken of two Ad.cre/cardiotoxin-induced mice with minimal HO growth (*red boxes*: zoomed regions of injection sites) at day 22. (D) High resolution microCT image of both injected limbs and representative cross sections depicting mineral deposition in day 22 Ad.cre/cardiotoxin-induced mouse (*red box*: imaged for cross sectional detection of HO from proximal (*top image*) to distal (*bottom image*) regions of growth; *red arrow*: plane of injected limb imaged and magnified to view minimal HO growth).

Since soft tissue infiltration causes decreased range of motion and other complications, the necessity of detecting HO at its early stages is apparent. In order to compare radiographic sensitivity, plain film radiographs were taken from mice forming small amounts of HO in our burn/tenotomy and Ad.cre/cardiotoxin-induced HO models at day 24 and day 22 respectively (Fig 5A and 5C). While these results were ambiguous, *in vivo* microCT scans of these same limbs clearly showed HO formation at earlier timepoints in both models (Fig 5B and 5D). This detection was further confirmed by microCT cross sectional imaging at sites of interest.

### MicroCT allows for quantification of additional HO bone metrics

Additional bone metrics such as tissue mineral density or porosity are often utilized to study normal bone development. To characterize these structural properties of HO over time, mice from our burn/tenotomy HO model were imaged at two timepoints. Tissue mineral density (TMD) of the tibia and HO in two anatomic locations was calculated, confirming the visual observations that regular bone structures are significantly more dense than ectopic bone (p<0.05), even at late timepoints (Fig 4A and 4B). Next, we assessed the porosity of the ectopic bone and found the HO porosity ratio to be significantly higher (p<0.001) than that of tibial bone across both timepoints (Fig 4C). When comparing 5 and 9-week values calculated for soft tissue HO porosity, we found the 5 week value to be significantly higher (p<0.05), suggesting a dramatic change in the soft tissue HO marrow space during the 5–9 week interval. Finally, we segregated the total HO volume into ectopic shell and trabecular volumes using manual and semi-automated methods, showing comparable values with both techniques (Fig 4D). Additional scans were performed on treated burn/tenotomy mice at 9 weeks to compare bone metrics of regular HO to that in mouse groups that were locally injected with celecoxib or apyrase. The same TMD analysis was applied to the HO in treatment groups and, as might be expected, revealed lower density values (Fig 4F). This was confirmed by cross sectional imaging (Fig 4F). Porosity was also calculated in treated HO groups showing higher values than that of untreated HO, once again illustrating the inverse relationship that exists between TMD and porosity as calculated here (Fig 4G).

## Discussion

Here we demonstrate the pitfalls associated with plain film radiographic imaging of heterotopic ossification (HO) and demonstrate the versatility of microCT imaging for HO evaluation. Although microCT has become a cornerstone of bone research[15–18], plain film radiography persists for studies of HO. Those studies that do utilize microCT are limited in their reporting of outcomes, focusing on total volume but neglecting other important aspects of HO including porosity or tissue mineral density. Because many studies of HO are centered on therapeutic interventions, understanding the effect of these interventions on bone parameters such as thickness, density, and trabecular space may yield important information to help guide therapy and even elucidate physiologic processes behind HO. Finally, serial microCT scans allow for precise measurements of changes in HO volume which can guide our understanding of treatment schedules and drug dosing.

To clearly illustrate the benefits of microCT imaging over plain film radiography, we evaluated three different models–burn/tenotomy, Ad.cre/cardiotoxin-induction, and genetic HO. Not only did microCT elucidate detailed visual data; we were also able to accurately and reproducibly distinguish HO growth from the native bone. This ability to manipulate the scan, creating 3-dimensional ROI’s as needed, allows for the determination of total and density-dependent volumes. Since marrow space is radiolucent, total volume cannot be determined with plain film radiography as measurements only reflect the quantity of the HO’s ectopic shell. But with microCT, researchers can evaluate HO total volume and then can use analysis parameters (800HU, 1250HU, and 1800HU thresholds are shown throughout this study) to automatically exclude the bone’s trabecular space from the measurement. Furthermore, this density-dependent isolation allows for TMD, porosity, and other bone measurements that can all be used to thoroughly characterize HO progression and structure. In our analysis of the literature, we found that several groups use collagen sponges with bone morphogenetic protein (BMP) impregnation[10]. The sponge can then be implanted intramuscularly in a location distant from the nearby anatomic bone. In this situation microCT is useful to allow for quantification in addition to clearly delineating the HO lesion from the normal bone. Because HO can be generated from periosteal outgrowth or within soft tissues, separate from bone, determining the spatial relationship between HO and normal bone which can obscure HO identification is critical.

Tissue mineral density (TMD) refers to a mass of bone matrix with specified density (evaluated here with 800HU and 1250 thresholds) that is divided by its volume. Alternatively, porosity indicates how much void space exists within the HO. Since HO is so variable in its formation, porosity can provide a standardized means for assessing the structural compression of the HO shell and marrow space. Earlier, we showed that TMD increased while porosity decreased with time (Fig 4A and 4C). This was supported by visual data and asserts the idea that as a piece of HO matures, the ectopic shell will condense and thicken while the trabeculae become more defined and segmented (Fig 4B). With advanced techniques in microCT analysis, it is now possible to quantify this anomaly, characterizing HO progression in a detailed, standardized manner. This ability was also illustrated when evaluating potential HO treatment groups, showing subtle but distinct differences in TMD and porosity outcomes (Fig 4E–4G).

While quantification of HO growth is important for outcome evaluation of experiments, it is important to note that other advanced imaging techniques may also improve our understanding of HO development. For example, magnetic resonance imaging (MRI) allows for identification of soft tissues with high water content, a key identifying factor for cartilage[20]. In fact, MRI is used in the clinical setting to identify cartilage defects and may be used to identify HO, a lesion that forms through endochondral ossification, prior to ossification[3,21,22]. MRI may also have potential in detecting changes regular bone morphology induced by HO[23,24]. Similarly, positron emission tomography (PET) scans allow for identification of foci with high metabolic activity[25]. Again identification of highly metabolic foci may allow us to determine regions that are destined for HO formation, even prior to cartilage deposition. Finally, Raman spectroscopy is an emerging tool that allows the identification of mineral deposition within tissues, which is central to the ossification process[26–28]. This technique has previously been used to detect early HO onset within a tendon transection model as early as 5 days post-injury[28].

MicroCT is not without its drawbacks however. It should be noted that analysis of microCT images requires a trained, dedicated user to ensure reproducible measurements. In addition, the files can often be large and require proprietary software for rendering and analysis. Both of these require a substantial amount of time especially when compared to plain film radiography. Furthermore, microCT is limited by the resolution which is obtained–higher resolution requires more electronic storage space and may be difficult to analyze without more advanced computers[17]. Additionally, other metrics to measure strength may not be provided by imaging techniques at all, but instead require functional assessments.

Although other imaging modalities may be useful for characterizing HO, microCT remains to be standard method for structural evaluation of the ectopic bone. Currently, the *de facto* standard for *in vivo* assessment of HO progression remains as volume quantification; only 6 studies (21%) that utilized microCT presented additional bone measurements. However, microCT imaging allows for a comprehensive volumetric quantification of the HO, which when coupled with TMD, porosity, shell, and trabecular measurements, can more precisely evaluate the progression of HO. For the clinician, our findings suggest that cross-sectional imaging such microCT is important in assessing the growth of HO in a patient, and for determining the location of the HO with relation to other critical structures such as joint spaces. Additionally, microCT imaging may provide clues as to why the patient is experiencing the symptoms associated with ectopic bone. Finally, due to the high recurrence rates associated with HO, it is important to fully delineate the extent of the HO to guide resection[29]. For the laboratory investigator, microCT is a powerful technique to analyze specimens; because samples are no longer available for structural analysis after decalcification and histologic processing it is prudent to obtain thorough data. However, microCT can be expensive and resource and time-intensive, and therefore thoughtful planning of experiments is required to ensure laboratory efficiency.

## Supporting Information

S1 Appendix List of published *in vivo* murine studies used to assess imaging techniques for HO. (PDF)
