# Supplementary material for: Characterization of Heterotopic Ossification Using Radiographic Imaging: Evidence for a Paradigm Shift
Source: PLoS One. 2015 Nov 6;10(11):e0141432. doi: 10.1371/journal.pone.0141432 (PMC4636348; doi:10.1371/journal.pone.0141432)
Supplement: S1 Appendix — (PDF) [file pone.0141432.s001.pdf]

Appendix

1.Ranganathan K, Peterson J, Agarwal S, Oluwatobi E, Loder S, Forsberg JA, Davis TA, Buchman SR, Wang SC, Levi B. Role of gender in burn-induced heterotopic ossification and mesenchymal cell osteogenic differentiation. Plast Reconstr Surg. 2015 Jun;135(6):1631-41. doi: 10.1097/PRS.0000000000001266.

2. Anthonissen J, Ossendorf C, Hock JL, Ritz U, Hofmann A, Rommens PM. A new small-animal model for the study of acquired heterotopic ossification after hip surgery. Acta Orthop Traumatol Turc. 2015;49(2):197-202. doi: 10.3944/AOTT.2015.14.0271.

3. Jaffe DE1, Yoo D, Blevins J, Gasbarro G, Hughes T, Paryavi E, Nguyen T, Fourney WL, Pellegrini VD Jr. Does Blast Medium Affect Heterotopic Ossification in a Blast-amputation Model? Clin Orthop Relat Res. 2015 Apr 28. [Epub ahead of print].

4. Pavey GJ, Qureshi AT, Hope DN, Pavlicek RL, Potter BK, Forsberg JA, Davis TA. Bioburden Increases Heterotopic Ossification Formation in an Established Rat Model. Clin Orthop Relat Res. 2015 Mar 31. [Epub ahead of print].

5. Polfer EM, Hope DN, Elster EA, Qureshi AT, Davis TA, Golden D, Potter BK, Forsberg JA. The development of a rat model to investigate the formation of blast-related post-traumatic heterotopic ossification. Bone Joint J. 2015 Apr;97-B(4):572-6. doi: 10.1302/0301-620X.97B4.34866.

6. Wang L, Carroll DO, Liu X, Roth T, Kim H, Halloran B, Nissenson RA. Effects of blockade of endogenous Gi signaling in Tie2-expressing cells on bone formation in a mouse model of heterotopic ossification. J Orthop Res. 2015 Mar 13. doi: 10.1002/jor.22876. [Epub ahead of print].

7. Qureshi AT1, Crump EK, Pavey GJ, Hope DN, Forsberg JA, Davis TA. Early Characterization of Blast-related Heterotopic Ossification in a Rat Model. Clin Orthop Relat Res. 2015 Mar 10. [Epub ahead of print].

8. Genêt F, Kulina I, Vaquette C, Torossian F, Millard S1, Pettit AR, Sims NA, Anginot A, Guerton B, Winkler IG, Barbier V, Lataillade JJ, Le Bousse-Kerdilès MC, Hutmacher DW, Levesque JP. Neurological heterotopic ossification following spinal cord injury is triggered by macrophage-mediated inflammation in muscle. J Pathol. 2015 Jun;236(2):229-40. doi: 10.1002/path.4519. Epub 2015 Mar 26.

9. Shi W, Xiao H, Xue F, Wu J. [Dynamic changes of matrix metalloproteinase 9 in heterotopic ossification of rat model]. Zhongguo Xiu Fu Chong Jian Wai Ke Za Zhi. 2014 Sep;28(9):1133-8.

10. Kang H, Dang AB, Joshi SK, Halloran B, Nissenson R, Zhang X, Li J, Kim HT, Liu X. Novel mouse model of spinal cord injury-induced heterotopic ossification. J Rehabil Res Dev. 2014;51(7):1109-18. doi: 10.1682/JRRD.2014.01.0019.

11. Zhang J, Zhao Y, Hou X, Chen B, Xiao Z, Han J, Shi C, Liu J, Miao Q, Dai J. The inhibition effects of insulin on BMP2-induced muscle heterotopic ossification. Biomaterials. 2014 Nov;35(34):9322-31. doi: 10.1016/j.biomaterials.2014.07.056. Epub 2014 Aug 15.

12. Peterson JR, Eboda ON, Brownley RC, Cilwa KE, Pratt LE, De La Rosa S, Agarwal S, Buchman SR, Cederna PS, Morris MD, Wang SC, Levi B. Effects of aging on osteogenic response and heterotopic ossification following burn injury in mice. Stem Cells Dev. 2015 Jan 15;24(2):205-13. doi: 10.1089/scd.2014.0291.

13. Perosky JE, Peterson JR, Eboda ON, Morris MD, Wang SC, Levi B, Kozloff KM. Early detection of heterotopic ossification using near-infrared optical imaging reveals dynamic turnover and progression of mineralization following Achilles tenotomy and

burn injury. J Orthop Res. 2014 Nov;32(11):1416-23. doi: 10.1002/jor.22697. Epub 2014 Aug 2.

14. Wu J, Xiao H, Xue F, Shi W, Zhao H. [Effects of selective and non-selective cyclooxygenase 2 inhibitors on heterotopic ossification in rat model with Achilles tenotomy]. Zhongguo Xiu Fu Chong Jian Wai Ke Za Zhi. 2014 Mar;28(3):371-6.

15. Werner CM, Zimmermann SM, Würgler-Hauri CC, Lane JM, Wanner GA, Simmen HP. Use of imatinib in the prevention of heterotopic ossification. HSS J. 2013 Jul;9(2):166-70. doi: 10.1007/s11420-013-9335-y. Epub 2013 Jun 21.

16. Kan L, Mutso AA, McGuire TL, Apkarian AV, Kessler JA. Opioid signaling in mast cells regulates injury responses associated with heterotopic ossification. Inflamm Res. 2014 Mar;63(3):207-15. doi: 10.1007/s00011-013-0690-4. Epub 2013 Dec 11.

17. Liu X1, Kang H, Shahnazari M, Kim H, Wang L, Larm O, Adolfsson L, Nissenson R, Halloran B. A novel mouse model of trauma induced heterotopic ossification. J Orthop Res. 2014 Feb;32(2):183-8. doi: 10.1002/jor.22500. Epub 2013 Oct 17.

18. Regard JB1, Malhotra D, Gvozdenovic-Jeremic J, Josey M, Chen M, Weinstein LS, Lu J, Shore EM, Kaplan FS, Yang Y. Activation of Hedgehog signaling by loss of GNAS causes heterotopic ossification. Nat Med. 2013 Nov;19(11):1505-12. doi: 10.1038/nm.3314. Epub 2013 Sep 29.

19. Mu X, Usas A, Tang Y, Lu A, Wang B, Weiss K, Huard J. RhoA mediates defective stem cell function and heterotopic ossification in dystrophic muscle of mice. FASEB J. 2013 Sep;27(9):3619-31. doi: 10.1096/fj.13-233460. Epub 2013 May 23.

20. Peterson JR1, De La Rosa S, Sun H, Eboda O, Cilwa KE, Donneys A, Morris M, Buchman SR, Cederna PS, Krebsbach PH, Wang SC, Levi B. Burn injury enhances bone

formation in heterotopic ossification model. Ann Surg. 2014 May;259(5):993-8. doi: 10.1097/SLA.0b013e318291da85.

21. Zhang K, Wang L, Zhang S, Yu B, Liu F, Cui Z, Jin D, Bai X. Celecoxib inhibits the heterotopic ossification in the rat model with Achilles tenotomy. Eur J Orthop Surg Traumatol. 2013 Feb;23(2):145-8. doi: 10.1007/s00590-012-0944-9. Epub 2012 Feb 10.

22. Wang M, Abbah SA, Hu T, Toh SY, Lam RW, Goh JC, Wong HK. Minimizing the severity of rhBMP-2-induced inflammation and heterotopic ossification with a polyelectrolyte carrier incorporating heparin on microbead templates. Spine (Phila Pa 1976). 2013 Aug 1;38(17):1452-8. doi: 10.1097/BRS.0b013e31828a3504.

23. Peterson JR1, Okagbare PI, De La Rosa S, Cilwa KE, Perosky JE, Eboda ON, Donneys A, Su GL, Buchman SR, Cederna PS, Wang SC, Kozloff KM, Morris MD, Levi B. Early detection of burn induced heterotopic ossification using transcutaneous Raman spectroscopy. Bone. 2013 May;54(1):28-34. doi: 10.1016/j.bone.2013.01.002. Epub 2013 Jan 11.

24. Pignolo RJ, Xu M, Russell E, Richardson A, Kaplan J, Billings PC, Kaplan FS, Shore EM. Heterozygous inactivation of Gnas in adipose-derived mesenchymal progenitor cells enhances osteoblast differentiation and promotes heterotopic ossification. J Bone Miner Res. 2011 Nov;26(11):2647-55. doi: 10.1002/jbmr.481.

25. Kan L, Lounev VY, Pignolo RJ, Duan L, Liu Y, Stock SR, McGuire TL, Lu B, Gerard NP, Shore EM, Kaplan FS, Kessler JA. Substance P signaling mediates BMP-dependent heterotopic ossification. J Cell Biochem. 2011 Oct;112(10):2759-72. doi: 10.1002/jcb.23259.

26. Tannous O, Griffith C, O'Toole RV, Pellegrini VD Jr. Heterotopic ossification after extremity blast amputation in a Sprague-Dawley rat animal model. J Orthop Trauma. 2011 Aug;25(8):506-10. doi: 10.1097/BOT.0b013e31821f6265.

27. Salisbury E, Rodenberg E, Sonnet C, Hipp J, Gannon FH, Vadakkan TJ, Dickinson ME, Olmsted-Davis EA, Davis AR. Sensory nerve induced inflammation contributes to heterotopic ossification. J Cell Biochem. 2011 Oct;112(10):2748-58. doi: 10.1002/jcb.23225.

28. Lin L, Shen Q, Leng H, Duan X, Fu X, Yu C. Synergistic inhibition of endochondral bone formation by silencing Hif1α and Runx2 in trauma-induced heterotopic ossification. Mol Ther. 2011 Aug;19(8):1426-32. doi: 10.1038/mt.2011.101. Epub 2011 May 31.

29. Leblanc E, Trensz F, Haroun S, Drouin G, Bergeron E, Penton CM, Montanaro F, Roux S, Faucheux N, Grenier G. BMP-9-induced muscle heterotopic ossification requires changes to the skeletal muscle microenvironment. J Bone Miner Res. 2011 Jun;26(6):1166-77. doi: 10.1002/jbmr.311.

30. Rodenberg E, Azhdarinia A, Lazard ZW, Hall M, Kwon SK, Wilganowski N, Salisbury EA, Merched-Sauvage M, Olmsted-Davis EA, Sevick-Muraca EM, Davis AR. Matrix metalloproteinase-9 is a diagnostic marker of heterotopic ossification in a murine model. Tissue Eng Part A. 2011 Oct;17(19-20):2487-96. doi: 10.1089/ten.TEA.2011.0007. Epub 2011 Aug 2.

31. Shimono K, Tung WE, Macolino C, Chi AH, Didizian JH, Mundy C, Chandraratna RA, Mishina Y, Enomoto-Iwamoto M, Pacifici M, Iwamoto M. Potent inhibition of heterotopic ossification by nuclear retinoic acid receptor-γ agonists. Nat Med. 2011 Apr;17(4):454-60. doi: 10.1038/nm.2334. Epub 2011 Apr 3.

32. Olabisi RM, Lazard ZW, Franco CL, Hall MA, Kwon SK, Sevick-Muraca EM, Hipp JA, Davis AR, Olmsted-Davis EA, West JL. Hydrogel microsphere encapsulation of a cell-based gene therapy system increases cell survival of injected cells, transgene expression, and bone volume in a model of heterotopic ossification. Tissue Eng Part A. 2010 Dec;16(12):3727-36. doi: 10.1089/ten.TEA.2010.0234. Epub 2010 Sep 1.

33. Toom A, Suutre S, Märtson A, Haviko T, Selstam G, Arend A. Lack of a central role for osteoprogenitor cells from the femoral canal in heterotopic ossification of the hip: an experimental study in a rat model. J Bone Joint Surg Br. 2010 Feb;92(2):298-303. doi: 10.1302/0301-620X.92B2.22630.

34. Xue T, Mao Z, Lin L, Hou Y, Wei X, Fu X, Zhang J, Yu C. Non-virus-mediated transfer of siRNAs against Runx2 and Smad4 inhibit heterotopic ossification in rats. Gene Ther. 2010 Mar;17(3):370-9. doi: 10.1038/gt.2009.154. Epub 2009 Nov 26.

35. Lin L, Shen Q, Xue T, Yu C. Heterotopic ossification induced by Achilles tenotomy via endochondral bone formation: expression of bone and cartilage related genes. Bone. 2010 Feb;46(2):425-31. doi: 10.1016/j.bone.2009.08.057. Epub 2009 Sep 6.

36. Shimono K, Morrison TN, Tung WE, Chandraratna RA, Williams JA, Iwamoto M, Pacifici M. Inhibition of ectopic bone formation by a selective retinoic acid receptor alpha-agonist: a new therapy for heterotopic ossification? J Orthop Res. 2010 Feb;28(2):271-7. doi: 10.1002/jor.20985.

37. Xu JC, Wu T, Wu GH, Zhong ZM, Tang YZ, Chen JT. Leptin expression by heterotopic ossification-isolated tissue in rats with Achilles' tenotomy. Saudi Med J. 2009 May;30(5):605-10.

38. Kan L, Liu Y, McGuire TL, Berger DM, Awatramani RB, Dymecki SM, Kessler JA. Dysregulation of local stem/progenitor cells as a common cellular mechanism for heterotopic ossification. Stem Cells. 2009 Jan;27(1):150-6. doi: 10.1634/stemcells.2008-0576.

39. Lin L, Chen L, Wang H, Wei X, Fu X, Zhang J, Ma K, Zhou C, Yu C. Adenovirus-mediated transfer of siRNA against Runx2/Cbfa1 inhibits the formation of heterotopic

ossification in animal model. Biochem Biophys Res Commun. 2006 Oct 20;349(2):564-72. Epub 2006 Aug 23.
